# Revision Spinal Surgery for Posterior Migration of Tantalum Cage: Tips and Tricks

**DOI:** 10.7759/cureus.23794

**Published:** 2022-04-03

**Authors:** Bing Wui Ng, Azmi Baharuddin, Jin Aun Tan, Mohd Hisam Muhamad Ariffin

**Affiliations:** 1 Department of Orthopaedics and Traumatology, Hospital Canselor Tuanku Muhriz (formerly Universiti Kebangsaan Malaysia), Kuala Lumpur, MYS; 2 Department of Orthopaedics, Hospital Pakar Kanak-Kanak, Universiti Kebangsaan Malaysia, Kuala Lumpur, MYS; 3 Department of Orthopaedics and Traumatology, Faculty of Medicine, Hospital Canselor Tuanku Muhriz (formerly Universiti Kebangsaan Malaysia), Kuala Lumpur, MYS

**Keywords:** plif, tlif, revision surgery, posterior migration, tantalum cage

## Abstract

The porous property of tantalum metal coupled with its high frictional surface and biocompatibility has made it an ideal biomaterial to facilitate bony fusion. This biomaterial is not unfamiliar to surgeons as it has been utilized with good clinical outcomes in arthroplasty. The usage of tantalum cages in spine surgery has gained traction. Complications resulting from the use of tantalum cage in lumbar fusion surgery were rarely reported. Here the authors would present a case of revision spinal surgery where the tantalum cage underwent migration from the previous posterior lumbar interbody fusion surgery. We further discuss ways to prevent such complications, precautions, tips, and tricks that could help other surgeons while dealing with this complication.

## Introduction

A porous tantalum cage is an open-cell structure designed in a dodecahedron pattern to simulate the biological cancellous bone. Its high porosity allows bone and tissue in-growth to provide secondary long-term stability [1]. Studies have shown that bone in-growth activity is twice higher than bony in-growth on polyetheretherketone (PEEK) implants [2]. The tantalum cage has an elastic modulus close to biological cancellous bone. The porous surfaces create a high frictional property which provides high initial stability when placed against the endplate of the vertebra [3]. Published accounts of the use of tantalum metal cage in lumbar surgery trackback as early as 2009 where trabecular metal (TM) cage was utilized in anterior lumbar interbody fusion (ALIF) [4]. Implant subsidence has been reported by several studies and is more frequently noticed in posteriorly inserted TM cages, likely due to the small footprint [5]. Revision surgery for posterior lumbar interbody fusion (PLIF) cage malposition is technically challenging. Herein, we present a revision case of malposition of TM cage in a posterior lumbar interbody fusion surgery and describe the details, tips, and tricks in managing and preventing this rare complication.

## Case presentation

A 40-year-old man presented to our center with complaints of gradually worsening right lower limb numbness, radicular pain, and weakness. The pain radiated from the back along the right posterior thigh to the posterior calf and sole. The patient has a prior history of open posterior lumbar interbody fusion (PLIF) done three years ago in another center due to back pain. The patient was symptom-free for a few months after the index surgery; however, the pain and numbness gradually increased in intensity. No bladder and bowel dysfunction was noted.

**Figure 1:**
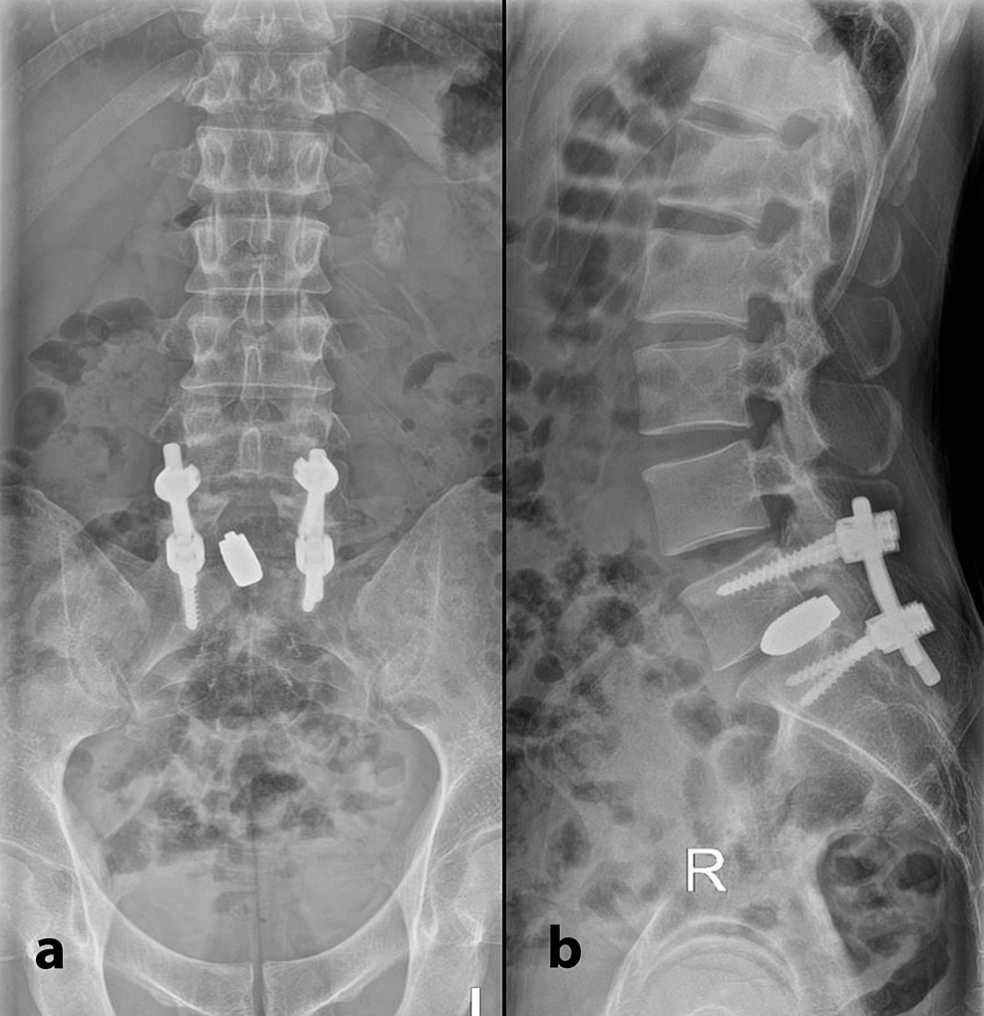
Antero-posterior (a) and lateral view (b) radiograph showing posterior instrumentation and malposition of tantalum cage protruding more than 50% into the spinal canal.

**Figure 2:**
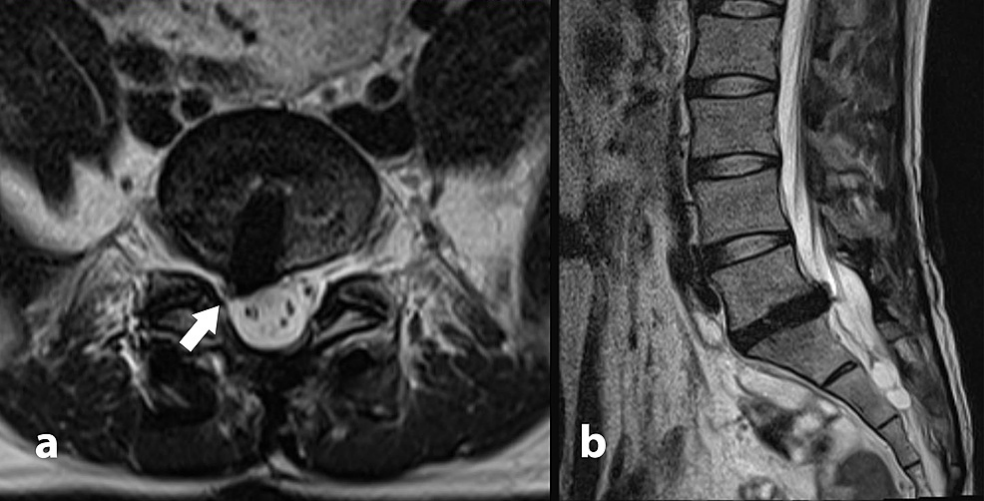
T2-weighted axial cut (a) and sagittal view (b) of MRI at L5/S1 level showing impingement of S1 traversing nerve root caused by the interbody tantalum cage. Arrow showing impingement of the tantalum cage on traversing S1 nerve root.

On examination, the patient’s lower limb tone was normal. The lower limb power of L5 and S1 is MRC grade 3. Loss of sensation at S1 dermatome of both feet. Radiological examination shows previous instrumentation and tantalum interbody cage at the L5/S1 disc. The tantalum cage was noted to be protruding into the spinal canal with the surrounding area of osteolysis (Figure 1). Magnetic resonance imaging (MRI) of the lumbar spine revealed impingement of the posterior aspect of the tantalum cage to the S1 traversing nerve root and abutment of the implant to the dura sac at the L5/S1 region (Figure 2). Revision surgery of removal of the interbody cage, exploration of the dura, and exiting nerve root with transforaminal interbody fusion (TLIF) was done for this patient.

Surgical technique

The patient was placed in a prone position on the spinal table under general anesthesia. The incision was made via the previous midline surgical incision. Scar tissue was noted as soon as we got near to the instrumentation. All pedicle screws and rods were removed. Larger pedicle screws of 6mm diameters were inserted in a more medialized fashion. Right facetectomy was done to allow an approach to the disc space. Abundant fibrous tissue was noted below the facet. Careful dissection was done until the posterior aspect of the tantalum cage was visualized. However, a sleeve of dura has in-grown into the porous surface of the tantalum cage, causing epidural fibrosis. The TM cage has incorporated well with the surrounding bone and has fibrous in-growth with the surrounding soft tissue. Unfortunately, the implant could not be removed without causing a dura tear.

**Figure 3:**
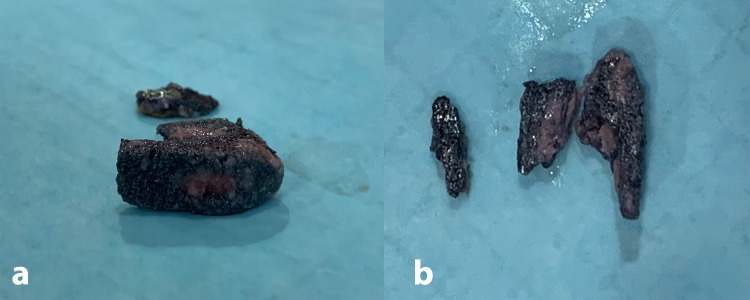
Clinical photo showing in-growth of fibrous tissue on the interbody cage (a) and trabecular metal interbody cage removed in three pieces (b).

First, the prominent posterior aspect of the implant was burred to flatten the surface. Saline was used to cooling the area to prevent thermal injury. The fragment of the implant with the dura in-growth was then removed, and the dura was protected with a nerve root retractor. The TM cage was then osteotomized in line with its porous surface to break it into smaller pieces to facilitate easier removal via the facetectomy window to prevent further injury to the dura and the surrounding vertebral endplate. The implant was then hammered into the disc space to disengage the implant-bone interface and later removed with a pituitary rongeur (Figure 3). The TM cage removed was size 9mm.

**Figure 4:**
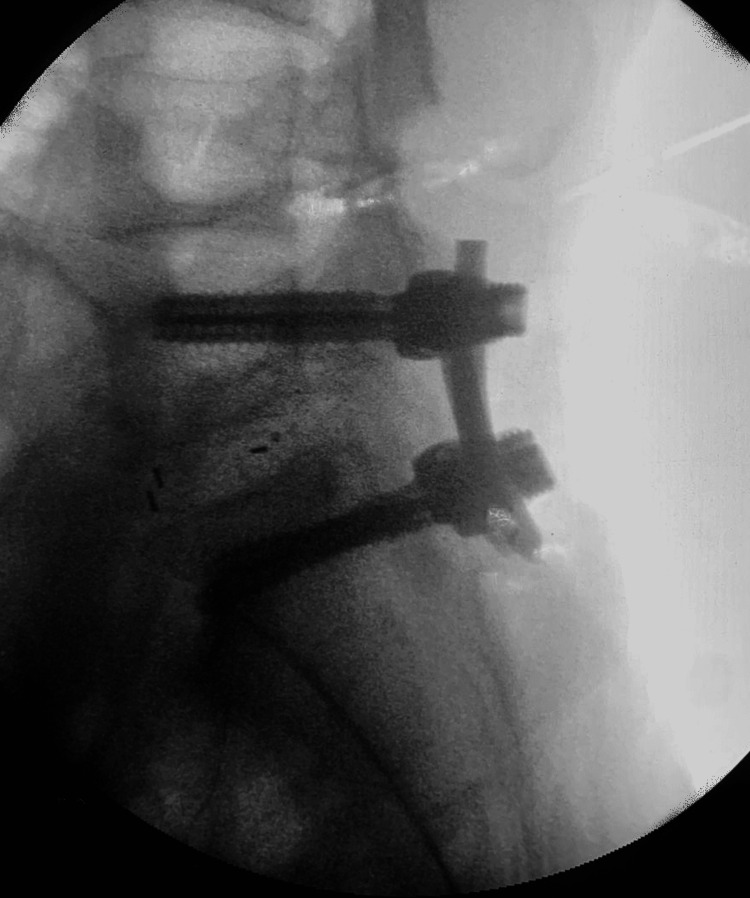
Immediate post-operative lateral view radiograph showing revision of spinal construct after removal of TM cage and insertion of TLIF PEEK cage.

Intraoperatively, the disc space was inspected, and a significant amount of disc material was further removed from the disc space. Preparation of the disc space was done with an endplate curette. The contralateral rod was placed to enable sequential dilatation of the disc space, and a size 11 PEEK cage was inserted under image intensifier guidance (Figure 4). The dura defect measured 2mm was closed with Surgicel (Oxidized regenerated cellulose; Johnson & Johnson Products, Inc., Patient Care Division, New Brunswick, NJ) and Tisseel (Baxter Healthcare Corp., Deerfield, IL).

Postoperatively patient’s radicular pain has resolved. The patient was followed up for nine months without any signs of recurrent pain and infection.

## Discussion

The insertion of a porous-surfaced TM cage with its high frictional properties is a challenging task. Soft tissues, exiting and traversing nerve roots have to be visually retracted far away from the margin of the path of cage insertion as any contact could produce significant injury to the dural sac causing cerebral spinal fluid (CSF) leakage. Thus, the pre-insertion preparation on the insertion path and disc space is of paramount importance to allow successful implantation. In this clinical case, the authors think that the previous surgeon had difficulty advancing the tantalum cage into the disc space due to inadequate preparation of anterior disc space, evident by the removal of disc material during the revision surgery. Besides that, inadequate distraction of the disc space by the dilator would make the insertion of the porous cage difficult due to its high frictional properties. Insertion of the implant size, which is the same size as the dilator, would prove more difficult. The authors think that due to these two points, the previous surgeon could have difficulty inserting the implant fully despite choosing a relatively small size 9 cage. The surrounding area of lucency visualized on the radiograph was not present in the MRI and clinical findings. Thus, we think that the cage was inadequately seated anteriorly.

Aoki et al., in their study, described that posteriorly migrated cage in PLIF could cause more symptomatic complications when compared to TLIF [6]. The paper also went on to suggest a more lateral insertion of the cage during the revision surgery to prevent further complications. Kimura et al. have described a pear-shaped L5/S1 disc space as a significant risk factor in causing cage retropulsion as the four corners of the cage do not engage the vertebral endplate uniformly, leading to instability [7]. Pan et al., in their study, concluded that early mobilization postoperatively could enhance local blood circulation promoting early fusion [8]. They also recommend that the revision surgery be done following the original invasion and to place the cages more laterally, further away from the neural elements. In this case, we have decided to use the same incision but convert the PLIF to TLIF to achieve objectives such as preventing excessive manipulation of epidural scar tissue, insertion of a more laterally directed interbody cage, and achieving better compression of the disc space facilitated by the unilateral facetectomy.

A few tips and tricks could be gathered from this literature. First, the interbody cage should be inserted with the disc space distracted to prevent damage to the body endplates, which in our case was facilitated by the sequential dilatation of the disc space using a dilator and by tightening the contralateral pedicle screws and rods. Second, compression of the pedicle screws and rod construct could further prevent any cage retropulsion. Third, the removal of disc materials and removal of cartilaginous layers of the endplates are paramount in ensuring good insertion and placement of the interbody cage. In our center, we routinely employ an exoscope to aid in better visualization of the anatomical structure. The good light source and optimal positioning of the exoscope enabled us to visualize the anterior annulus clearly to indicate adequate removal of disc material [9]. Fourth, do not choose undersized cages to prevent migration.

Removal of an in-grown porous tantalum cage is the real challenge in this case. To the authors' knowledge, no article has been written about the technique of removal of these cages. Surgeons attempting to take up these revision cages should anticipate dural leakage and the need for dural repair due to epidural fibrosis. We found that burr is useful in thinning the tantalum cage. Carbide tungsten burr tips could be used to break the implant into smaller removable fragments; however, we do not advocate this method for fear of the possible thermal damage, debris formation, and damage to the surrounding neural element. The use of small osteotome is useful in breaking the tantalum cage into small pieces. The direction of the cut should be aimed toward the anterior disc space to prevent soft tissue damage. Once the tip is sunk into the substance of the cage, a rotatory force could be introduced to break off the implant. Breaking the bone-implant interface is done by tamping the implant further into the disc space without cutting the bone-implant interface to prevent endplate fractures.

## Conclusions

This case serves as a reminder that the type of cages does not preclude the importance of adequate disc space and endplate preparation while performing spinal interbody fusion surgery. The authors intend to highlight the key points in the prevention of posterior migration of interbody cages, surgical planning in addressing a retro-pulsed porous tantalum cage, and tips and tricks for implant removal.
